# Human fetoplacental arterial and venous endothelial cells are differentially programmed by gestational diabetes mellitus, resulting in cell-specific barrier function changes

**DOI:** 10.1007/s00125-018-4699-7

**Published:** 2018-08-08

**Authors:** Silvija Cvitic, Boris Novakovic, Lavinia Gordon, Christine M. Ulz, Magdalena Mühlberger, Francisca I. Diaz-Perez, Jihoon E. Joo, Vendula Svendova, Michael G. Schimek, Slave Trajanoski, Richard Saffery, Gernot Desoye, Ursula Hiden

**Affiliations:** 1 Department of Obstetrics and Gynecology, Medical University of Graz, Auenbruggerplatz 14, 8036 Graz, Austria; 2 Cancer and Disease Epigenetics, Murdoch Children’s Research Institute, Royal Children’s Hospital, Melbourne, VIC Australia; 3 Institute for Medical Informatics, Statistics and Documentation, Medical University of Graz, Graz, Austria; 4 Center for Medical Research, Medical University of Graz, Graz, Austria

**Keywords:** Actin organisation, DNA methylation, Fetoplacental endothelial cells, Gestational diabetes mellitus, Programming

## Abstract

**Aims/hypothesis:**

An adverse intrauterine environment can result in permanent changes in the physiology of the offspring and predispose to diseases in adulthood. One such exposure, gestational diabetes mellitus (GDM), has been linked to development of metabolic disorders and cardiovascular disease in offspring. Epigenetic variation, including DNA methylation, is recognised as a leading mechanism underpinning fetal programming and we hypothesised that this plays a key role in fetoplacental endothelial dysfunction following exposure to GDM. Thus, we conducted a pilot epigenetic study to analyse concordant DNA methylation and gene expression changes in GDM-exposed fetoplacental endothelial cells.

**Methods:**

Genome-wide methylation analysis of primary fetoplacental arterial endothelial cells (AEC) and venous endothelial cells (VEC) from healthy pregnancies and GDM-complicated pregnancies in parallel with transcriptome analysis identified methylation and expression changes. Most-affected pathways and functions were identified by Ingenuity Pathway Analysis and validated using functional assays.

**Results:**

Transcriptome and methylation analyses identified variation in gene expression linked to GDM-associated DNA methylation in 408 genes in AEC and 159 genes in VEC, implying a direct functional link. Pathway analysis found that genes altered by exposure to GDM clustered to functions associated with ‘cell morphology’ and ‘cellular movement’ in healthy AEC and VEC. Further functional analysis demonstrated that GDM-exposed cells had altered actin organisation and barrier function.

**Conclusions/interpretation:**

Our data indicate that exposure to GDM programs atypical morphology and barrier function in fetoplacental endothelial cells by DNA methylation and gene expression change. The effects differ between AEC and VEC, indicating a stringent cell-specific sensitivity to adverse exposures associated with developmental programming in utero.

**Data availability:**

DNA methylation and gene expression datasets generated and analysed during the current study are available at the National Center for Biotechnology Information (NCBI) Gene Expression Omnibus (GEO) database (http://www.ncbi.nlm.nih.gov/geo) under accession numbers GSE106099 and GSE103552, respectively.

**Electronic supplementary material:**

The online version of this article (10.1007/s00125-018-4699-7) contains peer-reviewed but unedited supplementary material, which is available to authorised users.

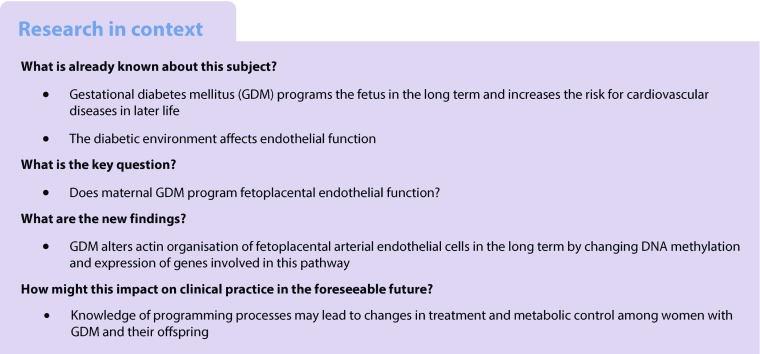



## Introduction

Adverse insults during intrauterine life can induce long-term changes in the physiology and metabolism of the offspring and, thus, program future health. This phenomenon is referred to as fetal programming [1, 2]. Epigenetic mechanisms, including DNA methylation, are characterised by a high degree of plasticity and responsiveness to environmental stimuli [3] and are considered to play a key role in fetal programming. Indeed, altered DNA methylation in response to the environment in utero leads to gene expression changes and consequently affects placental function [4, 5].

Gestational diabetes mellitus (GDM) is a pregnancy-related glucose intolerance causing maternal and fetal hyperglycaemia [6]. As with other types of diabetes, GDM is a growing problem and affects ~15% of pregnancies worldwide [7]. Although GDM resolves after birth, it is associated with long-term adverse consequences for mother and offspring (i.e. an increased risk for later diabetes in the mother and development of features of the metabolic syndrome, including cardiovascular disease, in the offspring) [8, 9].

Accumulating evidence linking intrauterine exposures to later risk of cardiovascular disease has raised interest in the process of vascular development and its programming by adverse maternal environments, such as GDM. Being directly exposed to the altered metabolic milieu of GDM, the endothelial compartment of the fetoplacental unit represents an ideal tissue for investigating the role of epigenetics due to adverse environments in programming cardiovascular/metabolic function [1, 10]. This notion is further supported by the observation that endothelial and vascular dysfunction [11] are among the most prominent long-term consequences of diabetes and hyperglycaemia.

The vascular system is comprised of two major types of blood vessels, arterial and venous, which differ in various physiological and anatomical factors, reflecting their distinct functions. The identity of arterial endothelial cells (AEC) and venous endothelial cells (VEC) is associated with distinct gene expression signatures established early in development [12]. Epigenetic mechanisms, including DNA methylation, are key to specifying such differences [13]. We previously demonstrated widespread DNA methylation differences in essential endothelial genes in fetoplacental AEC and VEC [14]. Importantly, the distinct phenotypical characteristics of these cells were maintained after isolation and during in vitro culture [15].

Here we hypothesised that the altered intrauterine environment associated with GDM modifies DNA methylation of endothelial cells of the fetoplacental unit in a cell-type-specific (AEC vs VEC) manner and aimed to identify the relationship between genome-wide methylation and gene expression in fetoplacental endothelial cells from healthy and GDM-complicated pregnancies. We conducted a pilot study to identify concordant gene methylation and expression changes induced by GDM, performed pathway analysis to identify functions and pathways subject to epigenetic dysregulation by GDM and tested a subset of these using functional studies.

## Methods

### Sample collection

Ethical approval was obtained from the Medical University Graz (25-008ex12/13 and 27-268ex14/15). All women participating in the study provided written informed consent. Control placentas were collected from pregnancies of non-smoking (self-reported) women who had a negative 75 g OGTT performed at 25–28 weeks of gestation and were free from medical disorders or pregnancy complications. GDM was diagnosed according to WHO/IADPSG criteria [16]. The women who had a positive OGTT were either recommended a diet and classified as GDM A1 or were additionally treated with insulin (NovoRapid plus Insulatard; Novo Nordisk Pharma, Vienna, Austria) and classified as GDM A2. Control and GDM samples were matched for ethnicity and fetal sex, but not for maternal BMI, since being overweight is major risk factor for GDM. However, neither BMI nor gestational weight gain differed between control and GDM groups. In addition, the mean and median cell passage of primary cells from control and GDM women was similar. We also performed covariate analysis on multiple factors (gestational age, cord blood insulin, fetal weight and length, fetal ponderal index, placental weight, maternal C-reactive protein [CRP], maternal height, maternal weight and BMI before pregnancy and before birth and maternal gestational weight gain) using Bioconductor package limma (https://bioconductor.org/packages/release/bioc/html/limma.html) and identified an effect on a total of 27 CpG sites at 21 genes (electronic supplementary material [ESM] Table 1), representing only a small subset of total differentially methylated positions and suggesting that observed effects are a result of GDM. We should note, however, that the sample size is too small for such covariate analysis.

We used different samples for expression and methylation analysis. Analysis of RNA and DNA from the same sample would have resulted in better correlation but analysis of different samples, though matched for clinical variables and passages, gives more robust results. Table 1 shows maternal, neonatal and placental characteristics of expression and methylation analyses.

**Table 1:** Maternal, neonatal and placental clinical variables for gene expression and DNA methylation analyses

Variable	Gene expression		DNA methylation	
	Normal pregnancy	GDM pregnancy	Normal pregnancy	GDM pregnancy
Maternal data				
No. of individuals used for cell isolation	8	14	9	9
No. of cell isolations (AEC/VEC)	8/8	10/11	9/9	9/5
Age (years)	34.0 ± 7.3	32.8 ± 7.9	28.4 ± 6.3	32.7 ± 6.6
Gestational age (weeks)	39.6 ± 1.5	38.9 ± 1.2	40.8 ± 1.5	39.2 ± 1.3*
Height (m)	1.66 ± 0.09	1.66 ± 0.06	1.70 ± 0.04	1.70 ± 0.05
Weight before pregnancy (kg)	77.3 ± 17.5	83.0 ± 16.6	75.0 ± 11.6	83.6 ± 18.5
BMI before pregnancy	27.7 ± 3.8	30.1 ± 5.6	25.8 ± 3.1	28.6 ± 7.6
Weight before birth (kg)	92.8 ± 20.0	95.1 ± 18.1	90.2 ± 11.5	94.6 ± 17.6
BMI before birth	33.3 ± 4.3	34.8 ± 5.8	30.1 ± 4.9	33.0 ± 6.6
Gestational weight gain (kg)	15.5 ± 4.2	12.1 ± 9.6	15.2 ± 2.7	10.9 ± 10.3
CRP (nmol/l)	99 ± 87	56 ± 49*	92 ± 88	54 ± 52
HbA_1c_ (mmol/mol)	ND	38.8 ± 2.2	ND	37.7 ± 2.3
HbA_1c_ (%)	ND	5.7 ± 0.2	ND	5.6 ± 0.2
OGTT glucose (mmol/l)				
Fasting	4.35 ± 0.44	5.11 ± 0.42*	4.35 ± 0.23	5.56 ± 0.66*
1 h	6.33 ± 0.96	10.20 ± 1.91*	6.23 ± 1.14	9.32 ± 2.33*
2 h	5.64 ± 0.63	7.05 ± 1.45*	5.33 ± 0.73	6.35 ± 1.54*
GDM classification (A1/A2)		9/5		4/5
Neonatal data				
Male/female ratio	4/4	6/8	4/5	4/5
Offspring weight (g)	3547 ± 428	3275 ± 409	3443 ± 482	3318 ± 365
Offspring length (cm)	51.0 ± 2.1	50.4 ± 2.0	50.4 ± 2.7	50.6 ± 2.7
Placental weight (g)	515 ± 190	620 ± 188	619 ± 195	595 ± 159
Fetal ponderal index	26.7 ± 2.0	25.5 ± 2.2	26.8 ± 2.6	25.9 ± 4.6
Fetus/placenta weight ratio	6.25 ± 1.37	5.72 ± 1.74	5.89 ± 1.44	6.02 ± 1.78
Cord blood insulin (pmol/l)	ND	223 ± 269	150 ± 177	181 ± 285
Cord blood C-peptide (nmol/l)	ND	1.06 ± 0.93	ND	0.60 ± 0.23
Cell culture data				
Cell isolation passage	6.1 ± 1.2	6.6 ± 1.1	5.4 ± 1.0	4.8 ± 0.6

Data are presented as means ± SD

The ethnicity was similar in all groups

*p < 0.05 by Student’s *t* test vs respective control group

ND, not determined

### Cell culture

Primary AEC and VEC were isolated from third-trimester human placentas after healthy and GDM-complicated pregnancies following a standard protocol [15]. Cells were characterised by immunocytochemical analysis [17] and cultured on 1% (vol./vol.) gelatin-coated flasks (75 cm^2^) using endothelial basal medium (EBM; Cambrex Clonetics, Baltimore, MD, USA) supplemented with the EGM-MV BulletKit (Cambrex Clonetics). Cell isolations were used up to passage ten as no phenotypical change or altered responses of the cells to culture were observed.

### DNA methylation analysis

DNA (1 μg) isolated from fetoplacental AEC (*n* = 9), VEC (*n* = 9), AEC from GDM pregnancies (dAEC) (*n* = 5) and VEC from GDM pregnancies (dVEC) (*n* = 9), obtained from nine control and nine GDM placentas in total, was bisulphite converted using MethylEasy Bisulphite Modification Kit (Human Genetic Signatures, Sydney, NSW, Australia). Conversion efficiency was assessed by bisulphite-specific PCR (not shown). Hybridisation of bisulphite-treated samples to Illumina Infinium Human Methylation450 (HM450) BeadChips (Illumina, San Diego, CA, USA) was performed according to the manufacturer’s instructions. Based on power calculations for the HM450 array, our sample size would allow us to detect changes of Δβ > 0.2 and *p* value <0.05 [18]. The BeadChips were scanned using Illumina iScan (Illumina) and raw data were exported as IDAT files. Minfi Bioconductor package (https://bioconductor.org/packages/release/bioc/html/minfi.html) [19] imported data into R (version R 2.15.1), performed quality control, pre-processing and normalisation using the subset-quantile within array normalisation (SWAN) method [20]. The limma package [21] was used to fit a linear model to compare dAEC and dVEC vs control samples, with patient as random effect and allowing for batch effects. False discovery rate (FDR) was calculated by the Benjamini–Hochberg method. *M* values were calculated after removing probes on the sex chromosomes to eliminate potential sex bias and poor-performing probes. β values were derived from intensities defined by the ratio of methylated (M) to unmethylated (U) probes given by β = M / (U + M + 100). For details of quality control, outlier identification and HM450 platform validation using locus-specific SEQUENOM MassARRAY EpiTYPER (Agena Bioscience, San Diego, CA, USA) [22], see ESM Methods and ESM Figs 1–3. For information on whether CpGs are located in differentially methylated regions (DMRs), see ESM Table 2 and for a discussion of advantages vs disadvantages of the HM450 platform, see ESM Methods. Unadjusted and adjusted *p* values and information on whether CpGs are located at potential SNPs, within topological domains (TADs) published in HUVECs [23] or part of a DMR, are given in the lists of significantly methylated CpGs available on Gene Expression Omnibus (GEO) database account GSE106099 (www.ncbi.nlm.nih.gov/geo).

### RNA isolation

Total RNA was isolated with RNeasy mini Kit (Qiagen, Hilden, Germany) and quality was assessed using a BioAnalyzer BA2100 (Agilent, Foster City, CA, USA) with the RNA 6000 Nano LabChip Kit (Agilent). Samples with an RNA Integrity Number ≥8.5 were further used.

### Microarray gene expression analysis

Total RNA from AEC (*n* = 8) and VEC (*n* = 8) isolated from eight placentas from normal pregnancies and dAEC (*n* = 11) and dVEC (*n* = 10) isolated from 14 placentas from pregnancies complicated by GDM was labelled using Ambion WT Expression Kit for Affymetrix GeneChip Whole transcript (WT) Expression Arrays (Life Technologies, Carlsbad, CA). The cRNA was hybridised to GeneChip Human 1.0 ST arrays according to the manufacturer’s instructions (Affymetrix, Santa Clara, CA, USA). Washing and staining (GeneChip HT Hybridization, Wash and Stain Kit; Affymetrix) was performed with Affymetrix GeneChip fluidics station 450. Arrays were scanned using Affymetrix GeneChip scanner GCS3000. Labelling and hybridisation controls were evaluated with Affymetrix Expression Console EC 1.1. Data were analysed with RMA (robust multi-chip average), including background correction, quantile normalisation, log_2_ transformation and median polish summarisation using Genomic Suite v6.5 (Partek, St Louis, MO, USA) [24]. Statistical analysis used one-way ANOVA with fetal sex and mother as random factors. The *p* values were adjusted for multiple testing using the Benjamini–Hochberg method (R/Bioconductor package ‘multtest’; https://www.bioconductor.org/packages/release/bioc/html/multtest.html) [25, 26].

### qPCR

cDNA was synthesized from 50 ng total RNA of different cell isolations (*n* = 10 per group) then used for microarray analysis according to protocol (SuperScript II Reverse Transcriptase protocol; Invitrogen, Carlsbad, CA, USA). qPCR was performed with TaqMan gene expression assays (Applied Biosystems, Carlsbad, CA, USA) and ABI Prism 5700 Sequence Detection System (Applied Biosystems, Foster City, CA, USA). The mean hypoxanthine-guanine phosphoribosyltransferase 1 (*HPRT1*) and ribosomal protein L30 (*RPL30*) expression was used as internal control as their expression was unaffected by GDM (not shown). Data were analysed using the ${2}^{-\Delta \Delta {\mathrm{C}}_{\mathrm{t}}}$ method [27]. Statistical analysis used Student’s *t* test in SigmaPlot (Systat Software, San Jose, CA, USA).

### Pathway analysis

Gene lists were analysed with Ingenuity Pathway Analysis (IPA, version 2.3) (Qiagen). Cut-off for methylation differences was *p* < 0.05 and Δβ ≥ 0.2 and for gene expression *p* < 0.05 and fold change (FC) ≥1.5. When investigating pathways the cut-off was set to Δβ ≥ 0.1 (methylation) and FC ≥1.3 (expression) in order to have sufficient genes for the analysis.

### F-actin immunofluorescence staining

AEC (*n* = 5), VEC (*n* = 6), dAEC (*n* = 5) and dVEC (*n* = 5), each in quadruplicates, were seeded in gelatin-coated chamber slides (50,000 cells/well). Participants’ characteristics are provided in ESM Table 3. After 24 h, slides were transferred to room temperature, washed with HBSS and fixed with 3.7% (wt./vol.) formaldehyde in PBS for 10 min. After washing with PBS, cells were permeabilised with 0.1% (vol./vol.) Triton X-100 in PBS for 25 min, washed with PBS, blocked with 1% (wt./vol.) BSA in PBS for 25 min and incubated phalloidin-488 FITC (1:20, Thermo Fisher, Eugene, OR, USA) with DL550 (DyLight 550 goat-anti mouse, 1:100, Thermo Fisher) for 1 h in the dark. Stained cells were washed with PBS and slides were mounted with Dako fluorescent mounting medium (Dako, Carpinteria, CA, USA) with DAPI (1:2000). After overnight drying, actin organisation was observed using a Zeiss LSM 510 Meta microscope, objective Plan-Apochromat 63×/1.4 Oil DIC, at 495 nm and 518 nm excitation wavelength (Zeiss, Oberkochen, Germany) using Zeiss LSM Image Browser. F-actin staining was performed and photographed by a blinded observer.

### Electrical cell-substrate impedance sensing

Impedance measurements were performed using an electrical cell-substrate impedance sensing (ECIS) system (Applied Biophysics, Troy, NY, USA) [28]. AEC (*n* = 10) and dAEC (*n* = 6) (80,000 cells/well), VEC (*n* = 8) and dVEC (*n* = 4) (110,000 cells/well) were seeded in 400 μl EBM on gelatin-coated gold electrodes (8W10E+ arrays; Applied Biophysics), in duplicates. Participants’ characteristics are provided in ESM Table 3. VEC are smaller in diameter and were seeded in higher density. Thus, both AEC and VEC reached confluency after 12 h. Impedance was then recorded at 4 kHz for 24 h. Linear mixed-effect model using R package nlme (https://CRAN.R-project.org/package=nlme) was fitted for AEC and VEC separately. GDM, time and the interaction between them were used as fixed effects. Time was also used as random effect.

## Results

### GDM alters the DNA methylation profile of fetoplacental AEC and VEC

**Fig. 1:**
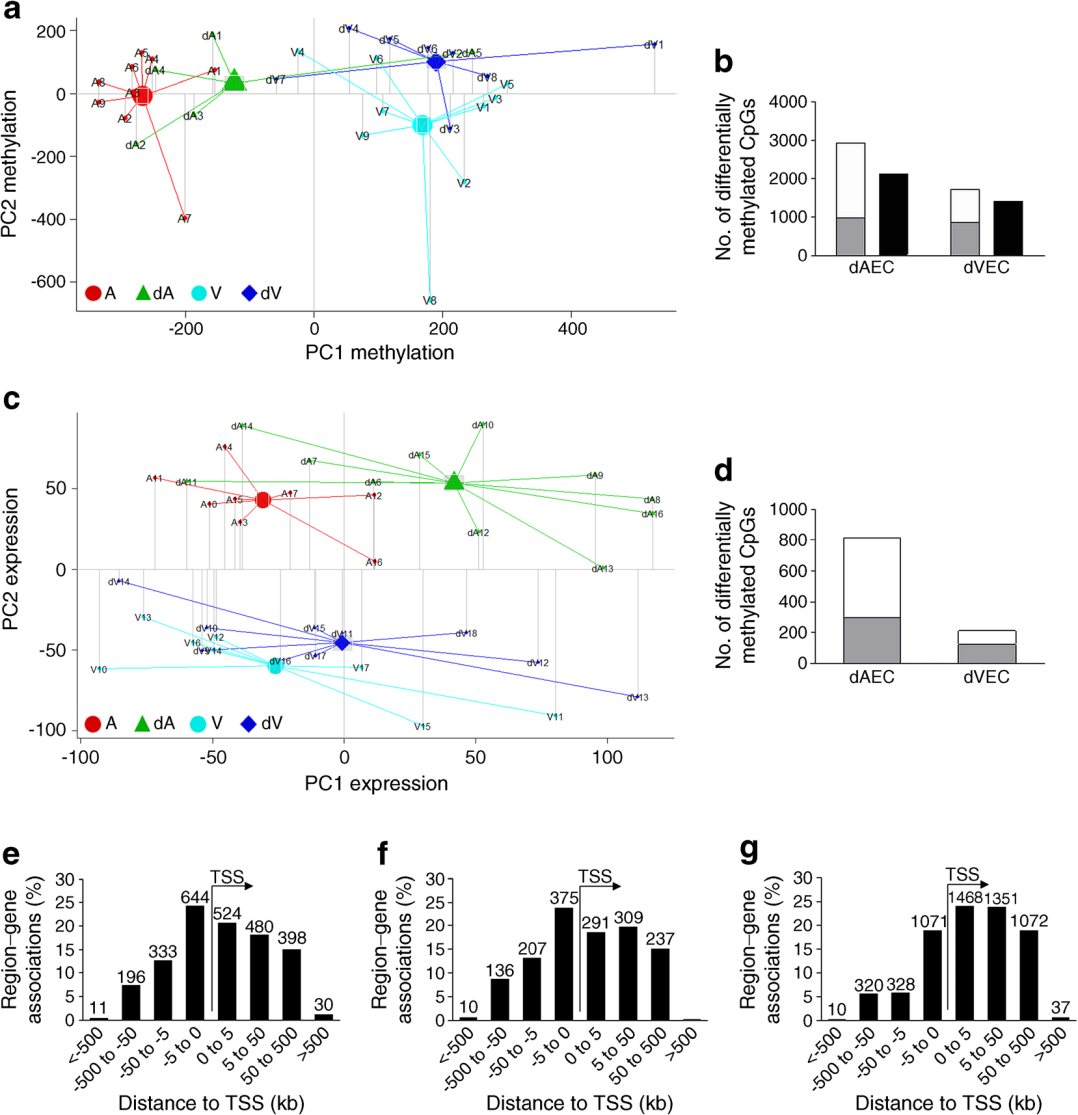
(**a**, **c**) PCA plot of DNA methylation (**a**) and gene expression (**c**) arrays. The first (PC1) and second principal components (PC2) are shown on the *x*- and *y*-axis, respectively. (**b**, **d**) Number of differentially methylated CpGs (**b**) and number of associated genes (**d**) in fetoplacental AEC and VEC exposed to GDM (dAEC and dVEC, respectively) vs control cells using a cut-off of *p* < 0.05, Δβ ≥ 0.2 for methylation and *p* < 0.05, FC ≥1.5 for gene expression. In (**b**), the grey section of the bars indicates the number of hypermethylated CpGs, the white section of the bars indicates the number of hypomethylated CpGs and the black bars indicate the number of associated genes. In (**d**), the grey section of the bars indicates the number of upregulated genes and the white section of the bars indicates the number of downregulated genes. (**e**–**g**) Great distance analysis of altered CpG methylation relative to transcription start site (TSS). Associations for gene regions are shown for differentially methylated (hyper- and hypomethylated) CpGs in dAEC vs AEC (**e**) and dVEC vs VEC (**f**) (*p* < 0.05, Δβ ≥ 0.2) and for differentially methylated CpGs between AEC and VEC (**g**) (*p* < 0.05, Δβ ≥ 0.4). The absolute number of associated genes is indicated above each bar

To analyse changes in DNA methylation profile associated with exposure of fetoplacental AEC and VEC to GDM in utero (dAEC and dVEC), we measured the methylation of >450,000 CpG sites. Principal component analysis (PCA) of the entire methylation dataset demonstrated cell type (AEC vs VEC) as a main source of variation but also showed clear separation according to GDM status (Fig. 1a). Hierarchical clustering identified cell type as the greatest contributor to methylation variation (ESM Fig. 1) while high correlation between technical replicates indicated low inter-array variability (ESM Fig. 2).

Linear regression indicated that exposure to GDM influenced methylation levels (*p* < 0.05 and Δβ ≥ 0.2) at 2617 CpGs that annotated to 2063 genes in dAEC and 1568 CpGs that annotated to 1360 genes in dVEC (Fig. 1b). The lists of differentially methylated CpGs are deposited as Excel files at GEO database, accession number GSE106099. Not surprisingly given the sample size, these sites did not remain significant following FDR adjustment for multiple testing applied to control for false discoveries. Of the total differential methylation associated with GDM, 351 genes and 51 CpGs were common to both cell types. In contrast to dVEC, where variation was equally distributed between hyper- and hypomethylation, dAEC showed hypermethylation at only 33% of sites while 67% were hypomethylated (Fig. 1b). This was also reflected by a marginal decrease of overall DNA methylation (average methylation levels detected across all probes) with GDM, particularly in AEC, as illustrated by kernel density plot and methylation index (ESM Fig. 4a, b). The box plots and heat maps shown in ESM Fig. 5 illustrate global and gene-specific methylation differences in GDM-exposed cells in more detail.

Great distance analysis showed that most of the GDM-associated variation in CpG methylation occurs in the proximal region upstream of genes (−5 to 0 kb from the transcriptional start) (Fig. 1e, f) in both cell types. In contrast to this, cell-type-specific methylation (AEC vs VEC) is enriched downstream of the transcription start site (0 to 50 kb; Fig. 1g).

Locus-specific DNA methylation profiling of selected genes significantly correlated with genome-wide measurements (*r*^2^ = 0.83 for arterial and *r*^2^ = 0.74 for venous group) and thus validated the HM450 DNA methylation platform (ESM Fig. 3).

### Pathways and functions of genes associated with altered methylation pattern in GDM

Functional analysis of differentially methylated genes in dAEC and dVEC revealed that the molecular functions ‘cellular function and maintenance’ and ‘cell morphology’ were predominantly altered. Unique and cell-specific affected functions with a high number of target genes were ‘cellular development’ in dAEC and ‘molecular transport’ in dVEC (Table 2).

**Table 2:** Top five significantly enriched molecular functions in Ingenuity Pathway Analysis for differentially methylated and top five for the differentially expressed genes in GDM-exposed AEC and VEC

Molecular function	*p* value	Score^a^
DNA methylation		
AEC		
Cellular function and maintenance	6.94 × 10^−6^ to 3.87 × 10^−2^	176
Cellular assembly and organisation^b^	2.86 × 10^−5^ to 3.87 × 10^−2^	114
Post-translational modification	1.54 × 10^−4^ to 3.77 × 10^−2^	26
Cell morphology^b^	2.39 × 10^−4^ to 4.01 × 10^−2^	92
Cellular development	2.39 × 10^−4^ to 4.40 × 10^−2^	147
VEC		
Cellular function and maintenance	8.24 × 10^−4^ to 4.91 × 10^−2^	53
Molecular transport	8.24 × 10^−4^ to 4.91 × 10^−2^	51
Cell morphology^b^	9.34 × 10^−4^ to 4.58 × 10^−2^	45
Cell-to-cell signalling and interaction	9.34 × 10^−4^ to 4.94 × 10^−2^	48
Cellular compromise	9.34 × 10^−4^ to 2.89 × 10^−2^	14
Gene expression		
AEC		
Cell cycle	8.42 × 10^−22^ to 1.26 × 10^−2^	159
Cellular assembly and organisation^b^	8.42 × 10^−22^ to 1.11 × 10^−2^	106
DNA replication, recombination and repair	8.42 × 10^−22^ to 1.11 × 10^−2^	159
Cellular movement^b^	2.54 × 10^−8^ to 1.11 × 10^−2^	89
Cellular growth and proliferation	1.03 × 10^−6^ to 1.15 × 10^−2^	234
VEC		
Cell cycle	1.75 × 10^−6^ to 1.83 × 10^−2^	48
Cell death and survival	4.96 × 10^−5^ to 1.36 × 10^−2^	25
Cellular assembly and organisation^b^	7.46 × 10^−5^ to 1.67 × 10^−2^	46
Cellular function and maintenance	7.46 × 10^−5^ to 1.67 × 10^−2^	38
DNA replication, recombination and repair	7.46 × 10^−5^ to 1.74 × 10^−2^	24

Genes significantly differentially methylated (*p* < 0.05; Δβ ≥ 0.2) or significantly differentially expressed (*p* < 0.05; FC ≥1.5) after GDM exposure in AEC and VEC, respectively, were enriched using Ingenuity Pathway Analysis. Molecular functions are comprised of multiple subcategories to which genes are assigned with different significance yielding a *p* value range (Fisher’s exact test)

^a^Score represents the number of affected genes assigned to a specific molecular function

^b^Molecular functions related to cell morphology and actin organisation

### GDM alters gene expression profile of fetoplacental AEC and VEC

PCA of the global transcriptome levels assessed at >30,000 transcripts in AEC and VEC clearly separated groups according to both cell type and GDM status (Fig. 1c). Unsupervised hierarchical clustering also discriminated AEC from VEC, again highlighting the greatest influence of cell type. Furthermore, dAEC vs control AEC clustered, while there was no clear separation in the venous group (ESM Fig. 6). Analysis of genes significantly influenced by GDM (*p* < 0.05) with a ≥ 1.5-fold expression change, identified 812 genes in dAEC, with 36% being upregulated and 64% downregulated (Fig. 1d). In dVEC only 211 genes had changed expression, with 57% being upregulated and 43% downregulated (Fig. 1d). Interestingly, only 92 genes were commonly affected by GDM in both cell types. A list of the top 100 differentially expressed genes in dAEC and dVEC vs controls is available at the GEO database, accession number GSE103552. A subset of differentially expressed genes identified by microarray was validated by qPCR, confirming differential expression of six out of nine genes (ESM Table 4).

### Pathways and functions of differentially expressed genes in GDM

Molecular functions enriched with the differentially expressed genes in GDM were ‘cell cycle’, ‘cellular assembly and organisation’ and ‘DNA replication, recombination and repair’ in both AEC and VEC (Table 2), indicating a strong common effect of diabetes.

### Concordant DNA methylation and expression changes in fetoplacental AEC and VEC

**Fig. 2:**
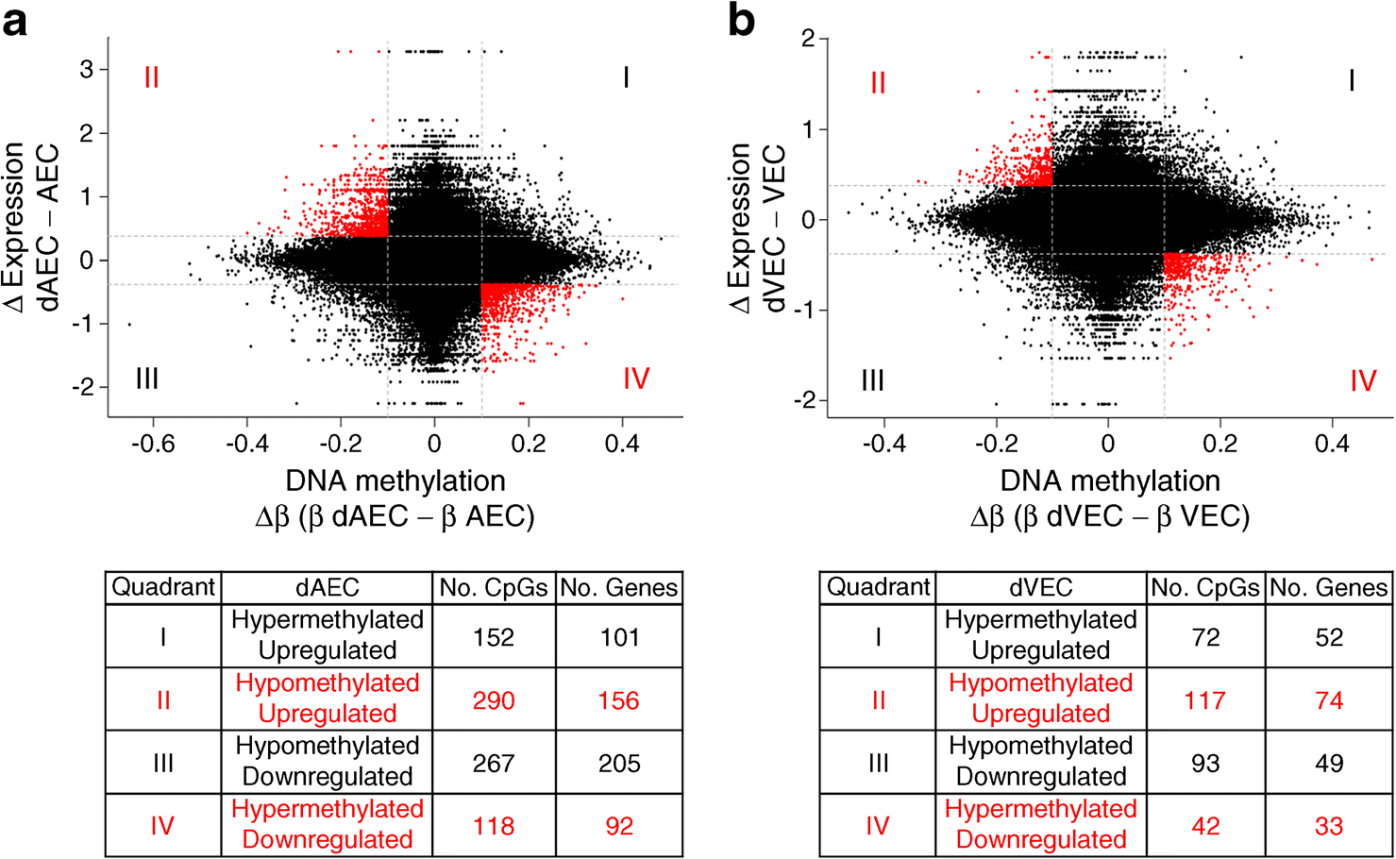
Relationship between DNA methylation and gene expression changes associated with GDM in fetoplacental AEC and VEC. Scatterplot of DNA methylation (*x*-axis) and gene expression (*y*-axis) for HM450 probes with differences between control and GDM-exposed AEC (**a**) and VEC (**b**). Cut-offs are set to ≥10% (Δβ ≥ 0.1) methylation difference and to FC ≥1.3 in the gene expression. Points in red indicate genes likely to be under epigenetic regulation by DNA methylation, with increased methylation associated with decreased gene expression and vice versa. The tables below the scatterplots indicate the respective direction of methylation and gene expression changes as well as the number of affected CpGs and genes

To investigate the relationship between DNA methylation and gene expression, methylation and gene expression datasets were combined. For each CpG, differences in β values between control cells and GDM-exposed cells (Δβ) were calculated and compared with corresponding gene expression differences. Setting the cut-off in the significant methylation difference to ≥10% (Δβ ≥ 0.1) and in the gene expression to FC ≥1.3, concordant changes in DNA methylation and gene expression (hypermethylated – downregulated and hypomethylated – upregulated) were detected. In dAEC, 118 genes were hypermethylated and downregulated, while 290 genes were hypomethylated and upregulated (Fig. 2a). In dVEC, 42 genes were hypermethylated and downregulated, while 117 genes were hypomethylated and upregulated (Fig. 2b). Of all genes showing concordant methylation and expression change in GDM, only six were altered in dAEC and dVEC (Table 3).

**Table 3:** Genes commonly regulated by GDM in AEC and VEC

Gene symbol	Gene name	Direction of expression and methylation change
*EGFR*	Epidermal growth factor receptor	Concordant in AEC and VEC Upregulated in AEC and downregulated in VEC
*GABBR1*	γ-Aminobutyric acid (GABA) B receptor, 1	Concordant in AEC and VEC Upregulated in AEC and VEC
*LMF1*	Lipase maturation factor 1	Concordant in VEC Upregulated in AEC and VEC
*NCKAP5*	NCK-associated protein 5	Concordant in AEC and VEC Upregulated in AEC and VEC
*SKIL*	SKI-like oncogene	Concordant in VEC Upregulated in AEC and VEC
*SULF2*	Sulfatase 2	Concordant in VEC Upregulated in AEC and VEC

The table shows genes significantly differentially methylated (*p* < 0.05; Δβ ≥ 0.1) and expressed (*p* < 0.05; FC ≥1.3) after GDM exposure in both AEC and VEC. Overlap was performed after integration of DNA methylation and gene expression data

Genes with concordant DNA methylation and gene expression change clustered to molecular functions involving actin reorganisation processes (‘cell morphology’ and ‘cellular movement’) in both dAEC and dVEC, with the largest number of affected molecules in ‘cell morphology’. ‘Carbohydrate metabolism’, ‘cell cycle’ and ‘cell-to-cell signalling and interaction’ were also listed as molecular functions significantly influenced by GDM in both cell types (Table 4).

**Table 4:** Top five significantly enriched molecular functions in Ingenuity Pathway Analysis for genes whose expression is potentially regulated by DNA methylation by GDM in AEC and VEC

Regulation	Molecular function	*p* value	Score^a^
AEC			
Hypomethylated/upregulated	Cell morphology^b^	5.79 × 10^−7^ to 1.63 × 10^−2^	40
	Cellular movement^b^	2.54 × 10^−5^ to 1.56 × 10^−2^	35
	Cell-to-cell signalling and interaction	5.75 × 10^−5^ to 1.63 × 10^−2^	28
	Carbohydrate metabolism	1.98 × 10^−4^ to 1.63 × 10^−2^	14
	Cellular development	1.98 × 10^−4^ to 1.63 × 10^−2^	33
Hypermethylated/downregulated	Cell-to-cell signalling and interaction	2.18 × 10^−5^ to 4.61 × 10^−2^	11
	Cellular compromise	2.18 × 10^−5^ to 1.82 × 10^−2^	10
	Molecular transport	5.86 × 10^−4^ to 4.74 × 10^−2^	10
	Cell cycle	7.69 × 10^−4^ to 4.86 × 10^−2^	11
	Cellular assembly and organisation^b^	7.69 × 10^−4^ to 4.61 × 10^−2^	16
VEC			
Hypomethylated/upregulated	Carbohydrate metabolism	4.55 × 10^−5^ to 1.17 × 10^−2^	4
	Small molecule biochemistry	4.55 × 10^−5^ to 2.72 × 10^−2^	11
	Cell morphology^b^	1.51 × 10^−4^ to 2.72 × 10^−2^	23
	Cellular function and maintenance	1.51 × 10^−4^ to 2.62 × 10^−2^	20
	Cell-to-cell signalling and interaction	1.58 × 10^−4^ to 2.72 × 10^−2^	11
Hypermethylated/downregulated	Cell cycle	1.48 × 10^−4^ to 4.85 × 10^−2^	4
	Protein synthesis	4.12 × 10^−4^ to 1.42 × 10^−2^	6
	Cellular movement^b^	4.76 × 10^−4^ to 4.58 × 10^−2^	3
	Cell death and survival	1.17 × 10^−3^ to 4.98 × 10^−2^	5
	Cell morphology^b^	1.42 × 10^−3^ to 4.71 × 10^−2^	4

Genes significantly differentially methylated (*p* < 0.05; Δβ ≥ 0.1) and differentially expressed (*p* < 0.05; FC ≥1.3) after exposure to GDM in AEC and VEC were enriched using Ingenuity Pathway Analysis. Molecular functions are comprised of multiple subcategories to which genes are assigned with different significance yielding a *p* value range (Fisher’s exact test)

^a^Score represents the number of affected genes assigned to a specific molecular function

^b^Molecular functions related to cell morphology and actin organisation

### Functional effects of GDM on actin organisation and barrier function

**Fig. 3:**
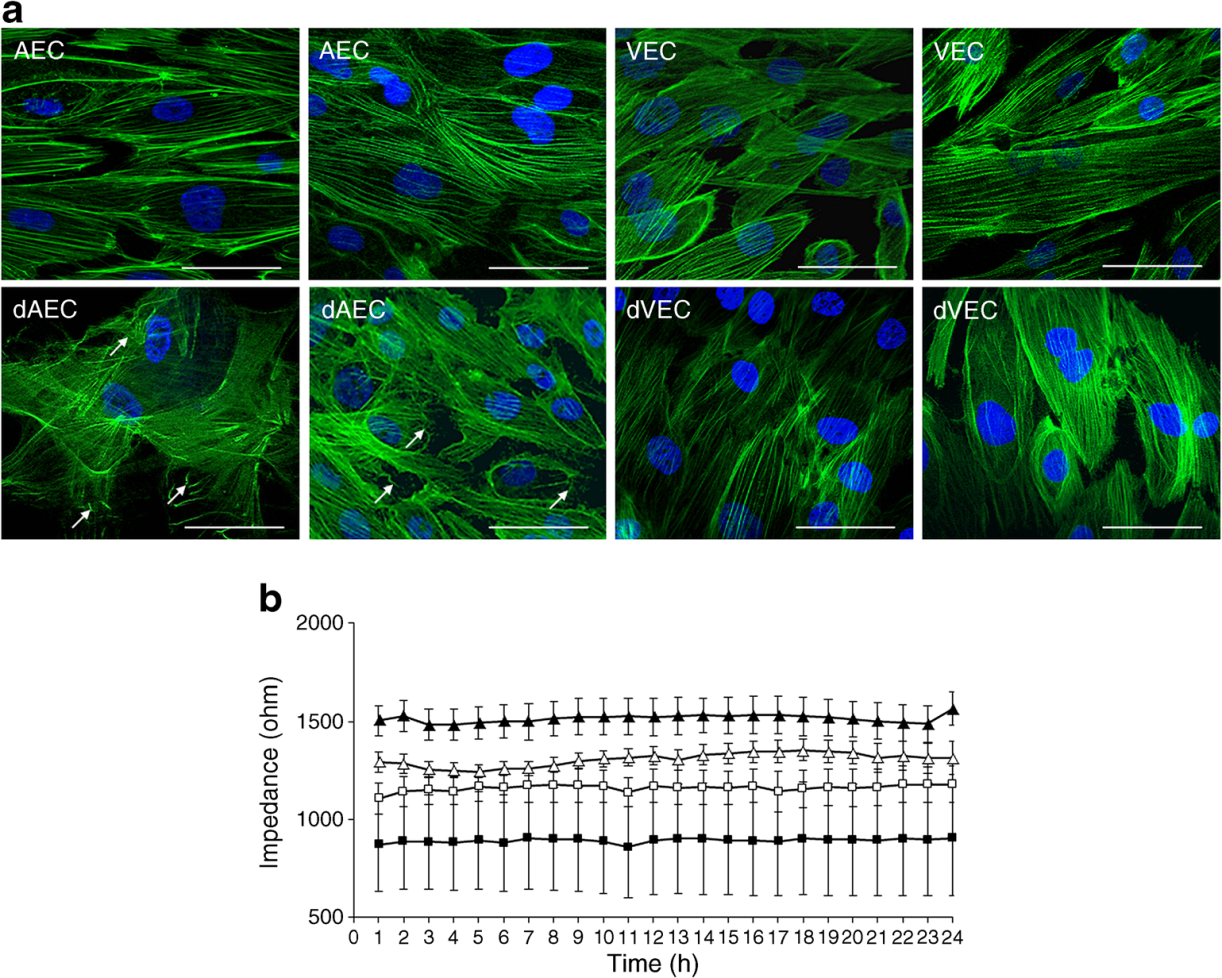
Effect of GDM on F-actin organisation and barrier function. (**a**) F-actin fibres in primary fetoplacental AEC and VEC, after normal or GDM pregnancy were stained with phalloidin (green). Nuclei were stained with DAPI (blue). Note that AEC after normal pregnancy show a more organised actin fibre network, while the dAEC, exposed to GDM, reveal more cross-linked F-actin bundles. This difference was not found in the dVEC, exposed to GDM. Two representative stainings of *n* = 5 (AEC and dAEC) or *n* = 6 individual cell isolations (VEC and dVEC) per group are shown, each performed in quadruplicate. Original magnification ×400. Scale bar, 40 μm. White arrows indicate less organised actin fibres in dAEC. (**b**) Barrier function was assessed by real-time analysis of the electrical impedance of cell monolayers. After reaching maximum impedance, endothelial resistance of primary AEC and VEC, after normal (*n* = 10 and 8, respectively) or GDM pregnancy (*n* = 6 and 4, respectively), was followed over 24 h. Data are plotted as means ± SEM at 1 h intervals. Data were generated in three independent experiments for each cell type separately, in duplicate. For statistical comparison, a linear mixed-effects model was fitted, showing significantly increased average impedance in dAEC vs AEC (*p* < 0.01). Reduced average electrical impedance of dVEC was not significant (*p* = 0.24). White triangles, AEC; white squares, dAEC; black triangles, VEC; black squares, dVEC

To link pathway analysis results to cellular function, we investigated in vitro actin organisation in control and GDM-exposed cells. Phalloidin staining of F-actin (Fig. 3a) demonstrated that exposure to GDM differentially disrupted actin organisation in AEC. While in control AEC the actin cytoskeleton was organised in parallel actin bundles, the cytoskeleton was disorganised in dAEC. In VEC, GDM did not induce similar effects.

As the actin cytoskeleton is essential for maintaining the endothelial barrier, we further investigated barrier function. Real-time analysis of the electrical impedance of cell monolayers showed an increased average impedance of 16% in dAEC vs AEC (*p* = 0.01) (Fig. 3b). In contrast, GDM tended to reduce the average impedance of VEC by 30%, but without reaching significance (*p* = 0.24). These distinct effects of GDM on barrier function in AEC and VEC parallel the aforementioned differences in actin organisation.

### Expression profile of genes involved in endothelial cell adhesion mirrors the effect of GDM on barrier function in fetoplacental AEC vs VEC

**Fig. 4:**
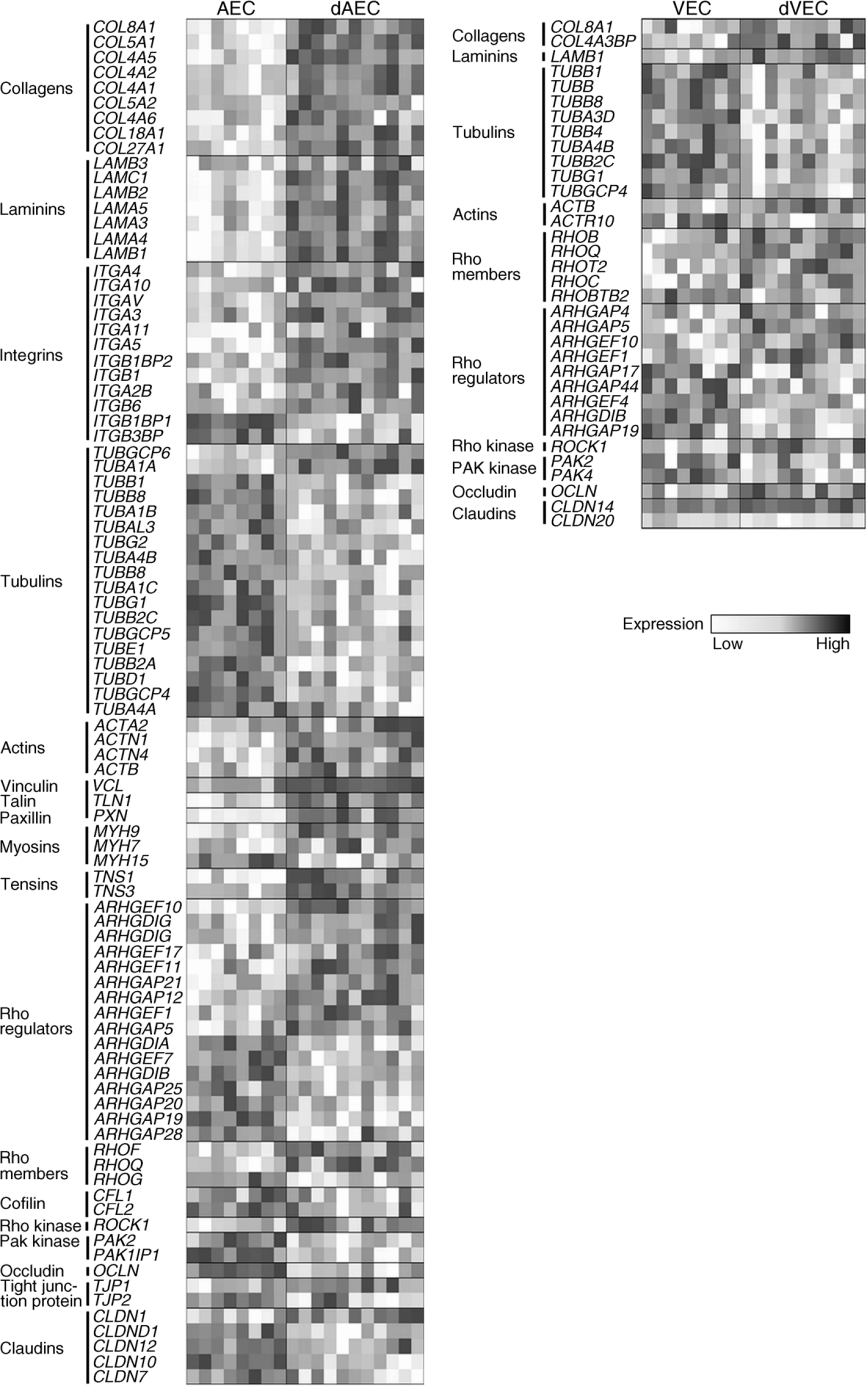
Heat map of genes implicated in endothelial cell barrier function. The heat map depicts only genes that show significant expression difference (*p* < 0.05) in GDM-exposed endothelial cells vs control cells and that are related to focal adhesion, adherens and/or tight junctions, or cytoskeleton actin organisation. The colour bar indicates the expression of a particular gene from low (white) to high expression (black)

Intercellular junction proteins join vascular endothelial cells and proteins of adherens junctions are most important for barrier function. Moreover, focal adhesions anchor endothelial cells to the basement membrane. Linker proteins such as vinculin and paxillin interconnect focal adhesions and intercellular junctions through the cytoskeleton. Their complex interplay in regulating actin organisation and vascular permeability is controlled by Rho GTPases [29]. We used expression levels of genes involved in focal adhesions, endothelial junctions and cytoskeleton rearrangement to generate a heatmap illustrating the effects of GDM on their expression in fetoplacental AEC and VEC (Fig. 4). This analysis revealed that GDM had the greatest effect on AEC, particularly on expression of focal adhesion-related genes: GDM upregulated various cellular integrins and extra cellular matrix genes, such as collagens and laminins, reflecting increased barrier function of dAEC potentially as a consequence of enhanced attachment to extracellular matrix. Rho GTPases and their upstream regulators (i.e. Rho guanine nucleotide exchange factors) and downstream targets (i.e. *ROCK1*) were also altered in dAEC. Fewer of these genes were affected by GDM in VEC.

## Discussion

The placenta is the largest vascular bed of the fetus. It is sensitive to exogenous exposures, reflected by the influence of diverse environmental stimuli on placental epigenetic marks, gene expression and function [5, 30]. The effect of GDM on DNA methylation has previously been investigated in total placental tissue [31–33]. However, the presence of distinct cell types complicates interpretation and calls for cell-type-specific approaches [5]. Furthermore, studies on the link between GDM-altered DNA methylation and resulting phenotypes have generally been limited to selected genes [34, 35]. The present study is the first attempt to delineate the effects of GDM on DNA methylation, transcription changes and functional consequences in offspring endothelium at the cellular level.

We report evidence that metabolic disturbances associated with GDM: (1) alter global DNA methylation and the gene expression profile of AEC and VEC; (2) have a cell-type-specific effect, with a greater number of genes affected in AEC than in VEC; and (3) affect genes involved in cell morphology, including those inducing cell-type-specific responses in actin organisation and barrier function.

GDM indeed altered DNA methylation and the gene expression profile of both AEC and VEC, supporting the concept of fetal metabolic programming by environmental stimuli. The individual signatures altered by GDM, however, differed between cells of arterial vs venous origin. Of the genes responsive to in utero exposure to GDM (as reflected by changes in DNA methylation and expression), only six were common to both AEC and VEC. These included *EGFR* and *SULF2*, encoding epidermal growth factor receptor and sulfatase 2, respectively, both linked to diabetic complications [36, 37]. The small number of genes commonly dysregulated by GDM in AEC and VEC strongly argues for cell-type-specific responses and metabolic programming of endothelial cells to the intrauterine environment. Indeed, VEC were less responsive to the GDM environment, as reflected by fewer CpGs and genes being affected, as compared with AEC. The reason for these cell-specific differences in susceptibility towards the GDM environment is unknown. Our previous studies showed that VEC are characterised by lower overall DNA methylation and a more juvenile phenotype than AEC [14, 15]. From this we speculated that VEC will be more amenable to the GDM-associated alterations. Moreover, differences in the two vascular loops with regard to the composition of cord blood constituents, blood flow and degree of oxygenation may play a role. Importantly, the venous blood composition of the fetoplacental unit depends on net exchange of available substrates between mother and placenta, while arterial blood composition is influenced foremost by fetal metabolism [38, 39]. The intrinsic factors in parallel with the metabolic disturbances in arteries and veins in GDM [40, 41] may account for differences and susceptibility towards GDM programming. It has yet to be discovered which of the factors altered in GDM may be responsible for altered DNA methylation and subsequent programming of cell function. Although transient hyperglycaemia is sufficient to induce permanent epigenetic changes in endothelial cells [42], the potential contribution of other factors should not be neglected. Based on pathway analysis of concordantly regulated genes, we identified ‘cell morphology’ as a pathway altered by GDM in both AEC and VEC. However, a cell-type-specific effect of GDM on programming of AEC and VEC morphology was found by studying actin organisation, which was influenced foremost in AEC. This arterial–venous distinction in response to GDM was maintained when we analysed barrier integrity, which also depends on actin organisation: cell monolayer impedance was increased in GDM-exposed AEC, paralleled by an upregulation of genes involved in endothelial cell adhesion. Our finding that GDM environment affects endothelial cell morphology through DNA methylation is supported by a previous study reporting that pathways related to ‘focal adhesion’ were influenced by GDM in cord blood and placenta in women of South Asian origin [33]. Moreover, ‘cell adhesion’ was reported to be the most enriched cluster of GDM-affected genes in the placenta [32].

The precise consequences that altered actin organisation may have on endothelial cell function of macrovascular vessels in vivo can only be hypothesised. The actin cytoskeleton drives the adaption to shear stress and optimisation of cell shape in response to blood flow [43, 44]. Moreover, actin organisation and its tethering to adhesion complexes is essential for endothelial barrier function [45] and fluid infiltration, to which macrovascular endothelial cells significantly contribute [46].

We acknowledge several limitations of our study such as the small sample size. As in other omics studies with small sample size [26], none of the differential methylation remained statistically significant following FDR adjustment for multiple testing. This, however, does not exclude the potential for phenotype-specific associations but additional evidence is required to support a link. The main aim of this study was to identify biologically relevant functions rather than individual genes affected by GDM and we believe our strategy of using less-stringent criteria for pathway analysis is appropriate. Furthermore, probes with unadjusted *p* values of <0.05 were chosen and validated using other techniques, for both the Affymetrix and HM450 platforms. We further recognise that we have no information on the composition of cord blood or on vascular function in the offspring and future studies are needed in this regard.

We do not know whether the GDM-induced changes observed in AEC and VEC isolated from placenta are representative for the vasculature of other fetal organs. However, both are exposed to the same circulation, tempting us to speculate that this is a possibility. Support comes from studies in diabetic adults, which report disturbances in the expression of focal adhesion components [47] and integrins [48] which may influence endothelial cell shape and barrier function [49], in line with long-term effects of intrauterine programming by diabetic environment, at least for these specific processes. Further, in other adult tissues such as adipose, processes related to regulation of cytoskeleton and focal adhesions seem to be susceptible towards epigenetic regulation by diabetic environment [50].

In conclusion, this is the first study to identify cell-specific effects of GDM in human primary endothelial cells of arterial and venous origin from the same vascular loop. Furthermore, we are the first to link cell-type-specific genome-wide-scale DNA methylation and gene expression changes in GDM with a follow-up of functional consequences indicated by pathway analysis. The results of this study will hopefully stimulate further work associating programmed endothelial changes in the placenta with long-term vascular alterations in GDM-exposed offspring.

## Electronic supplementary material

ESM (PDF 592 kb)

## Data Availability

DNA methylation and gene expression datasets generated and analysed during the current study are available at the National Center for Biotechnology Information (NCBI) Gene Expression Omnibus (GEO) database (http://www.ncbi.nlm.nih.gov/geo) under accession numbers GSE106099 and GSE103552, respectively.

## Abbreviations

AEC: Arterial endothelial cells
dAEC: AEC from GDM pregnancies
DMR: Differentially methylated region
dVEC: VEC from GDM pregnancies
EBM: Endothelial basal medium
FC: Fold change
FDR: False discovery rate
GDM: Gestational diabetes mellitus
PCA: Principal component analysis
VEC: Venous endothelial cells
