# Supplementary material for: Human fetoplacental arterial and venous endothelial cells are differentially programmed by gestational diabetes mellitus, resulting in cell-specific barrier function changes
Source: Diabetologia. 2018 Aug 8;61(11):2398–411. doi: 10.1007/s00125-018-4699-7 (PMC6182654; doi:10.1007/s00125-018-4699-7)
Supplement: Supplementary file 1 — (PDF 592 kb) [file 125_2018_4699_MOESM1_ESM.pdf]

Electronic Supplemental Material

providing detailed information on quality control of methylation and gene expression arrays, and additional, alternative representations of data

to

Human Feto-Placental Arterial and Venous Endothelial Cells Are Differentially Programmed by Gestational Diabetes Mellitus Resulting in Cell-Specific Barrier Function Changes

Silvija Cvitic[1], Boris Novakovic[2], Lavinia Gordon[2], Christine M Ulz[1], Magdalena Mühlberger[1], Francisca I Diaz-Perez[1], Jihoon E Joo[2], Vendula Svendova[3], Michael G Schimek[3], Slave Trajanoski[4], Richard Saffery[2], Gernot Desoye[1*] and Ursula Hiden[1*]

[1]Department of Obstetrics and Gynecology, Medical University of Graz, Austria

[2]Cancer and Disease Epigenetics, Murdoch Children's Research Institute, Royal Children's Hospital, Melbourne, Australia

[3]Center for Medical Research, Medical University of Graz, Graz, Austria

[4]Institute for Medical Informatics, Statistics and Documentation, Medical University of Graz, Austria

[*]The authors contributed equally to this work.

Corresponding author: Ursula Hiden

Medical University of Graz, Department of Obstetrics and Gynecology, Auenbruggerplatz 14, 8036 Graz, Austria

E-mail: ursula.hiden@medunigraz.at

Phone: 0043-316-385-17837

ESM Methods

Quality control and control of the inter-array variability of the Illumina DNA methylation arrays

Quality control was carried out using the Bioconductor packages arrayQualityMetrics, limma and MissMethyl. Based on the results of the quality control and clustering analysis (ESM Fig. 1), sample dAEC_6 was identified as an outlier and was excluded from further analysis. In the cluster dendrogram four venous samples, dVEC_2, VEC_10, dVEC_3 and VEC_8, clustered separately forming an individual group, but as they passed all quality control tests they were included in sequent analyses.

To control for inter-array variability four technical replicates of sample dVEC_4 were used and compared pairwise. Technical replicates grouped appropriately in the clustering analysis and showed very good correlations in pairwise comparison scatter plots (ESM Fig. 2). For further analysis one randomly selected replicate was used.

Validation of DNA methylation analysis: Locus-specific DNA methylation

Illumina Infinium Human Methylation450 (HM450) DNA methylation platform was validated using locus-specific SEQUENOM MassARRAY EpiTYPER platform. Genes for validation were chosen based on the methylation change between normal and diabetic cells (Δβ) and the size of the differentially methylated region (number of differentially methylated adjacent CpGs). Primer pairs for amplification were designed using EpiDesigner Web tool (http://www. epidesigner.com/). The regions of the respective genes were targeted using the following primers: forward 5′ aggaagagagTTGTAATTAAGGTTGGGTGTGTTTT 3′ and reverse 5′ cagtaatacgactcactatagggagaaggctATTCAAAACTCAAAATCCTACCCTC 3′ for nitric oxide synthase trafficker (*NOSTRIN*), forward 5′ aggaagagagAGGAGGGTTTTTTGGTTATTTTTTT 3′ and reverse 5′ cagtaatacgactcactatagggagaaggctAAATACCACAACCCCCATTTTAC 3′ for caveolin 2 (*CAV2*), 5′ aggaagagagTTTATTTGGATGTTGAAGGAATTTT 3′ and reverse 5′ cagtaatacgactcactatagggagaaggctTTCTCAAAATAAACCAATACAAACC 3′ for DEAD (Asp-Glu-Ala-Asp) box polypeptide 60-like (*DDX60L*), forward 5′ aggaagagagGTGAATTTTTTTTGTGGGAATAATG 3′ and reverse 5′ cagtaatacgactcactatagggagaaggctACTAAAAAACTCTCTCCCCAACCTA 3′ for natriuretic peptide C (*NPPC*). Amplification was performed after bisulfite conversion of genomic DNA with the MethylEasyXceed bisulphite conversion kit (Human Genetic Signatures, North

Ryde, Australia). Amplification conditions were 40 cycles of 95°C for 5min, 56°C for 1min 30s and 72°C for 1 min 30s, and then 72°C for 7min.

Significant difference in methylation of one CpG within *CAV2* and one within *NPPC* gene investigated with HM450 was confirmed with MassARRAY system (ESM Fig. 5b and 5d). Although *NOSTRIN* and *DDX60L* did not show a significant methylation change with MassARRAY system in the CpGs that are in common with the HM450, the average methylation change in the respective gene region was significantly altered in GDM exposed cells (ESM Fig. 5a and 5c). Furthermore, methylation status of *NOSTRIN* and *CAV2* genes for AEC and *DDX60L* and *NPPC* genes for VEC obtained with HM450 significantly correlated with the methylation data from MassARRAY system thereby validating the HM450 platform (ESM Fig. 5e and 5f).

Advantages and Disadvantages of HM450K Illumina DNA methylation arrays

Genome-wide DNA methylation analysis always involves a trade-off between coverage and depth [1]. The 450K array covers most ENCODE assigned distal regulatory regions and gene promoters, however it covers a very small percentage of all CpG sites in the genome. Techniques like enhanced reduced representation bisulfite sequencing (ERRBS) and whole genome bisulfite sequencing (WGBS) cover a markedly higher number of CpG sites. In our study we expected small differences in DNA methylation, and therefore, chose a technique that is reasonably quantitative and more cost-effective. Future studies can consider more global screening approaches such as EPIC array (850K), ERRBS, or WGBS that offer more coverage and therefore provide information about CpG sites that are not covered by the 450K array [2].

References

[1] Laird PW (2010) Principles and challenges of genomewide DNA methylation analysis. Nat Rev Genet 11: 191-203

[2] Pidsley R, Zotenko E, Peters TJ, et al. (2016) Critical evaluation of the Illumina MethylationEPIC BeadChip microarray for whole-genome DNA methylation profiling. Genome Biol 17: 208

ESM Table 1. Genes comprising differentially methylated CpGs that revealed correlation in co-variate analysis testing. Testing was performed for gestational age, cord blood insulin, fetal weight and length, fetal ponderal index, placental weight, maternal CRP, maternal height, maternal weight and BMI before pregnancy and before birth.

**Clinical parameter**	**No of CpGs**	**Genes**
Maternal CRP	3	*BRD9, MICAL2*
Fetal ponderal index	8	*SNTB1, DEAF1, FGGY, ROR1, PKLR, VPS4A*
Fetal gender	8	*RFTN1, CSMD1, ALG11, UTP14C, DAZL, TAPBP*
Gestational age	8	*TUBA3E, PLEKHA7, LCN10, TNIP3, SEPT9, CARTPT, MYCBP*

No correlation was identified with cord blood insulin, fetal weight and length, placental weight, maternal height, maternal weight and BMI before pregnancy and before birth, and gestational weight gain.

ESM Table 2. Identification of differentially methylated regions (DMRs) with two or more differentially methylated positions (DMPs) in VEC vs sVEC (upper panel) and AEC vs dAEC (lower panel).

**Differential methylated CpGs in GDM exposed AEC**					
**Gene symbol**	**AEC methylation**	**dAEC methylation**	**Methylation change (Δβ)**	**DMR size (bp)**	**No. of differentially methylated CpGs**
PRDM9	0.365	0.126	-0.240	206	7
SAMD11	0.598	0.390	-0.208	631	5
CECR2	0.467	0.177	-0.289	188	5
CRTAC1	0.700	0.415	-0.286	1052	4
CUX2	0.746	0.486	-0.260	478	4
HMHA1	0.655	0.399	-0.256	377	4
VPS53	0.636	0.299	-0.337	252	4
UPP1	0.580	0.869	0.289	178	4
MEIS2	0.682	0.401	-0.281	145	4
TRIM60	0.707	0.408	-0.299	107	4
ZNF846	0.865	0.519	-0.346	434	3
CD1D	0.425	0.179	-0.246	403	3
ABCA13	0.472	0.159	-0.312	363	3
JAG2	0.573	0.810	0.237	307	3
ESYT3	0.141	0.422	0.281	290	3
ZFP57	0.381	0.326	-0.055	288	3
AKAP10	0.191	0.475	0.284	276	3
RBM46	0.527	0.287	-0.240	269	3
PNLDC1	0.778	0.542	-0.236	228	3
ZFP42	0.503	0.226	-0.276	224	3
DPYSL3	0.505	0.795	0.290	220	3
PIWIL3	0.649	0.322	-0.327	219	3
ATP6V1B1	0.319	0.579	0.260	218	3
SERP1	0.695	0.360	-0.335	206	3
SLC6A1	0.690	0.331	-0.360	200	3
FLJ42875	0.627	0.890	0.263	199	3
CACNB2	0.527	0.273	-0.254	180	3
DIO3	0.645	0.274	-0.371	112	3
GSTA4	0.421	0.721	0.300	109	3
HLA-DRB5	0.311	0.543	0.232	290	2
AFF3	0.441	0.180	-0.261	278	2
HTATIP2	0.374	0.630	0.257	266	2
LRBA	0.520	0.773	0.253	263	2
FAM150B	0.564	0.256	-0.309	252	2
UMODL1	0.626	0.412	-0.215	246	2
HSPA2	0.409	0.680	0.271	240	2
CBX5	0.298	0.615	0.316	232	2
NAV1	0.168	0.415	0.247	230	2
IRF5	0.398	0.638	0.241	226	2
AGBL1	0.384	0.173	-0.211	219	2
KLK15	0.539	0.263	-0.276	204	2

FLI1	0.660	0.904	0.244	203	2
KPRP	0.720	0.455	-0.265	195	2
NRN1	0.480	0.183	-0.297	191	2
FAM101A	0.309	0.629	0.320	185	2
CHL1	0.360	0.119	-0.241	179	2
GATA3	0.589	0.847	0.258	164	2
VWA3B	0.477	0.220	-0.257	160	2
FRMD4A	0.378	0.165	-0.213	159	2
NLGN1	0.309	0.554	0.244	154	2
CTNNA2	0.476	0.185	-0.291	146	2
AHNAK	0.410	0.729	0.319	144	2
NKX6-2	0.268	0.520	0.251	144	2
ZFPM1	0.457	0.770	0.313	119	2
COL21A1	0.348	0.612	0.264	116	2
NPHS2	0.648	0.417	-0.231	108	2
VAX2	0.584	0.874	0.290	106	2
NQO2	0.430	0.648	0.218	100	2
EIF5A2	0.362	0.122	-0.240	97	2
CD300A	0.200	0.428	0.229	96	2
FAAH	0.679	0.415	-0.265	94	2
ACADM	0.357	0.122	-0.235	94	2
ZNF836	0.292	0.620	0.328	90	2
SHOX2	0.288	0.309	0.021	90	2
RAI1	0.478	0.727	0.249	88	2
SOX6	0.508	0.791	0.283	87	2
HPGD	0.488	0.257	-0.231	87	2
GLRX3	0.684	0.435	-0.249	84	2
LHPP	0.482	0.715	0.233	81	2
SORL1	0.626	0.372	-0.253	81	2
CCNG2	0.619	0.347	-0.272	77	2
GRAMD1B	0.327	0.080	-0.248	77	2
CASZ1	0.274	0.608	0.335	75	2
FBXO4	0.720	0.279	-0.440	65	2
DES	0.293	0.519	0.226	63	2
PTPRN2	0.439	0.715	0.276	60	2
BRDT	0.897	0.676	-0.221	60	2
NXN	0.454	0.712	0.258	58	2
FAM20B	0.687	0.485	-0.203	58	2
C10orf108	0.573	0.251	-0.322	56	2
DPEP1	0.495	0.251	-0.244	54	2
ATP4B	0.448	0.201	-0.246	54	2
PTPRN2	0.773	0.553	-0.220	52	2
CTSF	0.212	0.465	0.253	49	2
ADCY7	0.562	0.785	0.223	46	2
SDR16C5	0.609	0.389	-0.219	45	2
RTP1	0.384	0.157	-0.227	43	2
CAMTA1	0.502	0.806	0.305	42	2
DHRS3	0.163	0.407	0.244	42	2
SLCO1B3	0.753	0.388	-0.365	37	2

ANGPT2	0.243	0.511	0.268	36	2
PDGFRA	0.249	0.453	0.203	34	2
TMEM232	0.282	0.073	-0.210	33	2
SPESP1	0.476	0.223	-0.253	30	2
HLA-DPB2	0.717	0.486	-0.230	29	2
BTNL2	0.642	0.337	-0.305	29	2
RASL10B	0.702	0.942	0.240	28	2
CDH12	0.420	0.110	-0.310	27	2
KCNA10	0.489	0.220	-0.270	22	2
LTBP1	0.435	0.703	0.268	21	2
GALNT3	0.382	0.667	0.285	21	2
DTNA	0.524	0.152	-0.372	21	2
EFCAB4B	0.667	0.908	0.241	19	2
KIF26A	0.617	0.390	-0.227	18	2
CAV1	0.272	0.524	0.252	17	2
BAIAP2	0.279	0.519	0.240	17	2
RAP1GAP	0.906	0.670	-0.236	15	2
ODZ3	0.786	0.522	-0.264	13	2
PRHOXNB	0.225	0.510	0.285	13	2
OCLN	0.719	0.466	-0.254	13	2
PRAME	0.670	0.383	-0.287	13	2
TBX15	0.494	0.703	0.209	12	2
HOXA4	0.400	0.663	0.263	12	2
ESR1	0.485	0.211	-0.274	10	2
CXCL16	0.237	0.488	0.251	9	2
CLU	0.507	0.711	0.205	7	2
OTUB1	0.504	0.230	-0.274	7	2
RAPGEFL1	0.460	0.681	0.220	6	2
MSLNL	0.834	0.556	-0.278	5	2
NCOA6	0.316	0.094	-0.222	4	2
WNT4	0.201	0.515	0.314	2	2
SV2B	0.169	0.405	0.236	2	2
TAL1	0.139	0.373	0.234	2	2

Differential methylated CpGs in GDM exposed VEC

Gene symbol	VEC methylation	dVEC methylation	Methylation change (Δβ)	DMR size (bp)	No. of differentially methylated CpGs
DDX60L	0.055	0.300	0.245	235	5
ABAT	0.518	0.290	-0.227	512	4
CCDC48	0.521	0.106	-0.415	110	3
DPP6	0.431	0.759	0.328	166	3
ENPP2	0.671	0.387	-0.283	247	3
GBX2	0.318	0.546	0.227	568	3
MAL2	0.386	0.702	0.316	146	3
MEIS1	0.320	0.562	0.242	249	3
ZBTB9	0.589	0.504	-0.085	170	3
ATP4A	0.396	0.402	0.006	62	2
BLCAP	0.802	0.574	-0.227	52	2
C1orf173	0.387	0.138	-0.249	91	2

C3orf27	0.299	0.525	0.225	7	2
C5orf47	0.788	0.580	-0.208	140	2
CDH20	0.840	0.577	-0.263	143	2
CR2	0.675	0.457	-0.218	149	2
GALNACT1	0.464	0.246	-0.219	46	2
CYP26A1	0.558	0.277	-0.281	181	2
FBXL18	0.311	0.611	0.300	12	2
GLI3	0.572	0.315	-0.257	172	2
HAMP	0.652	0.378	-0.274	11	2
IFITM1	0.133	0.336	0.203	180	2
IMPA2	0.248	0.504	0.256	61	2
IZUMO1	0.402	0.158	-0.244	187	2
LHX9	0.136	0.429	0.294	204	2
MIR196A2	0.686	0.389	-0.297	9	2
NKX6-2	0.512	0.298	-0.214	6	2
PDE4D	0.293	0.501	0.208	101	2
PIWIL2	0.746	0.464	-0.282	254	2
PLCB2	0.653	0.411	-0.243	14	2
RNF39	0.801	0.556	-0.244	209	2
SMOC2	0.441	0.711	0.269	71	2
SNTG2	0.636	0.341	-0.295	77	2
TNN	0.583	0.812	0.229	119	2
TNXB	0.720	0.472	-0.247	9	2
TRIM10	0.269	0.579	0.310	28	2
TUB	0.500	0.756	0.256	114	2
UCN3	0.494	0.805	0.311	12	2
UTS2	0.304	0.543	0.238	7	2
WNK4	0.185	0.413	0.228	80	2
ZMYND10	0.417	0.188	-0.229	6	2

ESM Table 3. Maternal, neonatal and placental clinical parameters for Phalloidin stainings and ECIS monolayer impedance measurements.

		**Actin organisation**		**Monolayer impedance**	
		Normal	GDM	Normal	GDM
Maternal data	Number of individual patients for cell isolation	10	9	12	10
	Number of cell isolations (AEC/VEC)	5/6	5/5	10/8	6/4
	Maternal age (yrs)	28.4±5.9	33.6±5.5	28.6±7.1	30.0±6.8
	Gestational age (wks)	38.6±1.1	39.0±0.9	40.0±1.6	39.1±0.9
	Maternal height (m)	1.66±0.06	1.67±0.04	1.68±0.07	1.62±0.07
	Weight before pregnancy (kg)	64.2±3.8	80.9±7.8*	70.6±12.3	70.9±25.1
	BMI before pregnancy	23.3±2.5	29.2±7.8*	24.8±3.8	26.8±8.5
	Weight before birth (kg)	71.8±7.3	87.8±12.2*	83.4±13.3	93.1±22.1
	BMI before birth	26.5±3.7	31.7±12.2	30.5±3.5	34.6±6.5
	Gestational weight gain (kg)	7.3±7.5	6.9±8.0	15.2±4.1	9.0±11.0
	CRP (nmol/l)	64±27	74±44	54±50	60±111
	HbA1c (mmol/mol)	n.d.	32.6±3.5	n.d.	34.9±3.9
	Fasting oGTT (mmol/l)	4.43±0.32	4.41±0.21*	4.40±0.41	5.08±0.39*
	1h oGTT (mmol/l)	5.39±1.42	11.04±1.83*	6.49±0.91	10.24±2.49*
	2h oGTT (mmol/l)	4.88±0.49	6.49±14.71*	5.33±0.72	7.04±1.92*
	GDM classification (A1/A2)		5/5		6/4
Neonatal data	Offspring weight (g)	3073±450	3388±243	3312±584	3260±308
	Offspring length (cm)	49.1±2.0	51.1±1.8*	49.6±2.0	49.7±1.8
	Placental weight (g)	602±195	621±146	637±222	620±109

	Fetal ponderal index	25.8±1.5	25.4±2.0	26.9±3.6	26.6±2.1
	Feto-placental weight ratio	5.35±1.49	5.54±1.04	5.36±2.42	5.28±0.67
	Cord blood insulin (pmol/l)	n.d.	109±92	n.d.	179±209
	Cord blood C-peptide (nmol/l)	n.d.	1.14±1.04	n.d.	1.42±1.08
	Cell isolation passage	7.7±1.3	8.0±1.6	7.9±1.4	7.7±1.9

The ethnicity was similar in all groups. All isolation derived from female placentas.

Data are indicated as mean±SD.

* $p < 0.05$ by students t-test vs respective control group.

BMI = body mass index

CRP = C-reactive protein

HbA1c = glycosylated hemoglobin

oGTT= oral glucose tolerance test

n.d. = not determined

ESM Table 4. Validation of genes that were differentially expressed in GDM exposed AEC vs. control in the microarray analysis.

Gene symbol	Gene name	Microarray		qPCR	
		**p-value**	**FC**	**p-value**	**FC**
VCAN	Versican	<0.001	9.74	<0.05	8.38
FBN1	Fibrillin 1	<0.001	3.05	<0.001	6.75
TGFBI	Transforming growth factor, beta-induced	<0.001	2.84	<0.05	2.42
CCND2	Cyclin D2	0.025	2.39	<0.05	4.11
IGF1R	Insulin-like growth factor 1 receptor	<0.001	1.67	<0.05	2.38
GADD45B	Growth arrest and DNA-damage-inducible, beta	0.001	1.35	0.063	1.95
p300	E1A binding protein p300	0.001	1.28	<0.001	3.57
GADD45A	Growth arrest and DNA-damage-inducible, alpha	0.04	1.18	0.067	1.80
FANCC	Fanconi anemia, complementation group C	<0.001	-1.31	n.s.	n.c.

Genes, whose expression was significantly regulated by GDM by a variable fold, were selected for validation by qPCR. For calculation of $2^{-\Delta\Delta ct}$ value, the geometrical mean of ct values of the housekeeping genes *RPL30* and *HPRT1* was used. All selected genes, except *GADD45A* (p=0.067), *GADD45B* (p=0.063) and FANCC (n.s.: not significant; n.c.: no change) reached statistical significance and were confirmed to be differentially expressed between control and GDM exposed cells, thus validating the microarray platform. FC (fold-change) is the ratio of mean expression for GDM exposed vs. control cells.

ESM Fig. 1

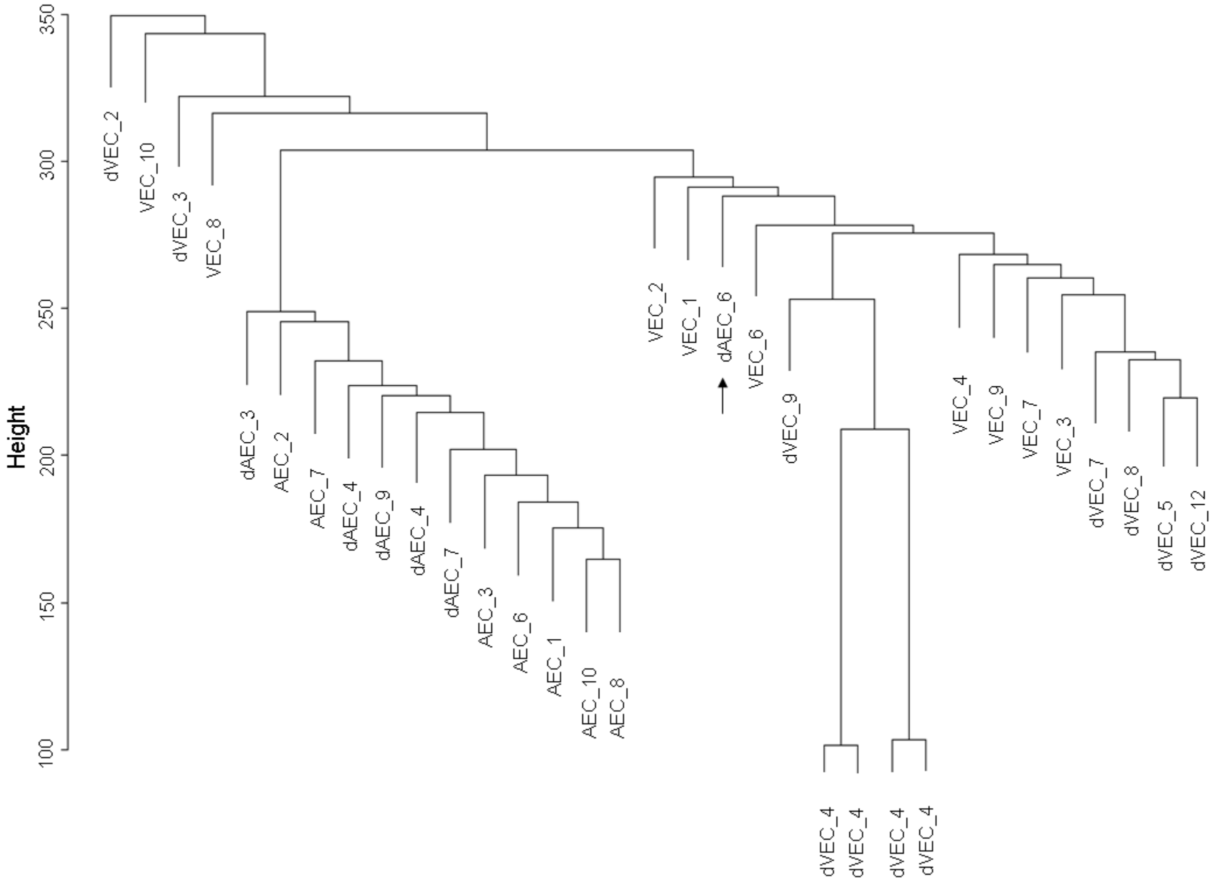

ESM Figure 1. Cluster dendrogram of methylation arrays showing influence of cell type and GDM on sample clustering. Arrow marks the arterial outlier sample dAEC_6 that clusters with venous samples. Rectangle indicates four technical replicates of sample dVEC_4 used to control for inter-array variability. Sample relations based on 426302 genes with SD/mean >0.1.

ESM Fig. 2

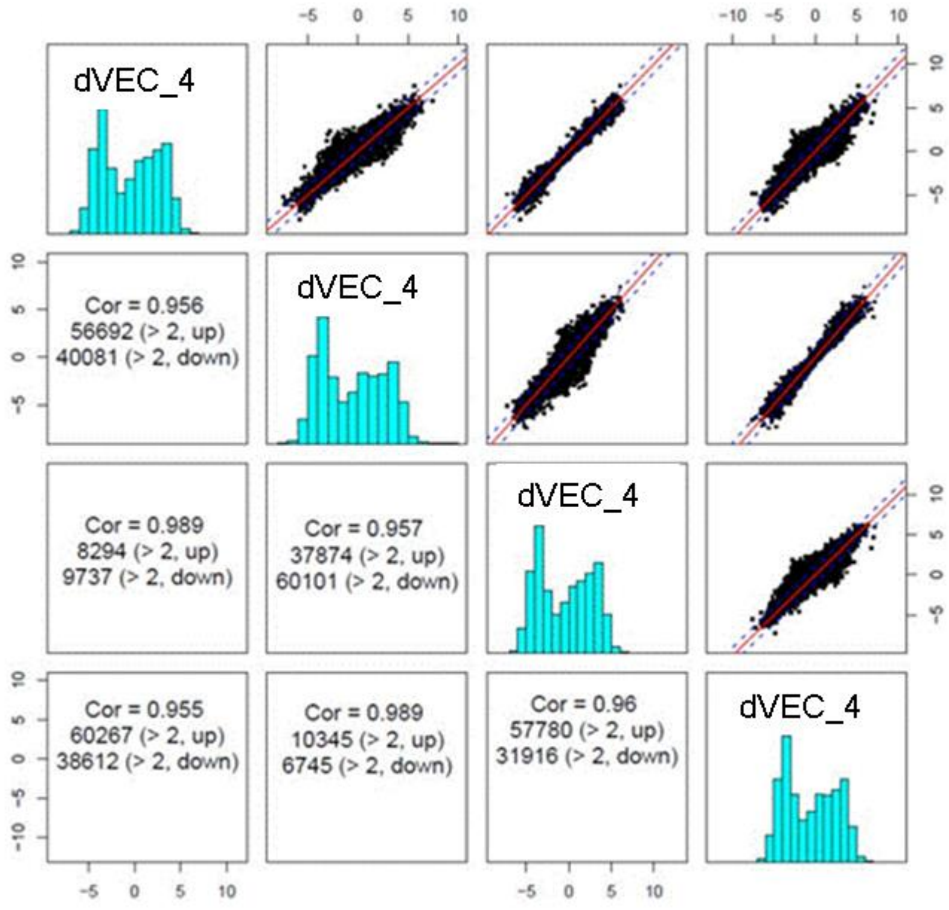

ESM Figure 2. Scatterplots of the four technical replicates of sample dVEC_4 were used to control for inter-array variability. Pairwise comparison with sample correlation was performed on all investigated CpG probes.

ESM Fig. 3

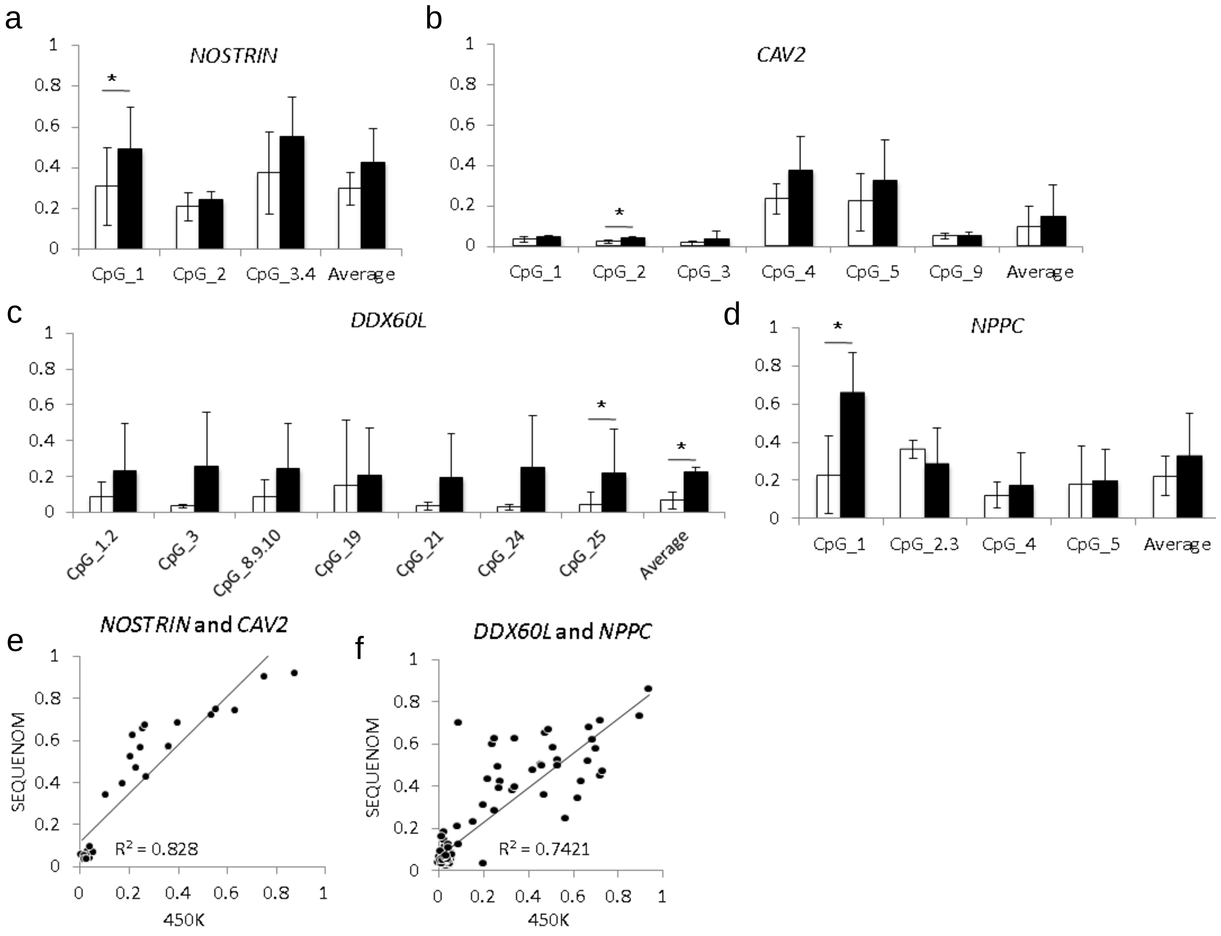

ESM Figure 3. Validation of Infinium HumanMethylation450 BeadChip arrays via MassARRAY system (SEQUENOM). *NOSTRIN* and *CAV2* were chosen for validation of methylation differences of AEC and *DDX60L* and *NPPC* of VEC exposed to GDM vs. control cells. Methylation status of individual CpGs from the MassARRAY measurement is shown. CpG2 and CpG3.4 for *NOSTRIN* (a), CpG2, CpG4, CpG5 and CpG9 for *CAV2* (b), CpG3, CpG21 and CpG25 *DDX60L* (c) and all shown CpGs for *NPPC* (d) are in common with the HM450. Methylation status of the chosen probes obtained with HM450 array significantly correlated ($p < 0.05$) with the methylation data from Sequenom (e, f).

ESM Fig. 4

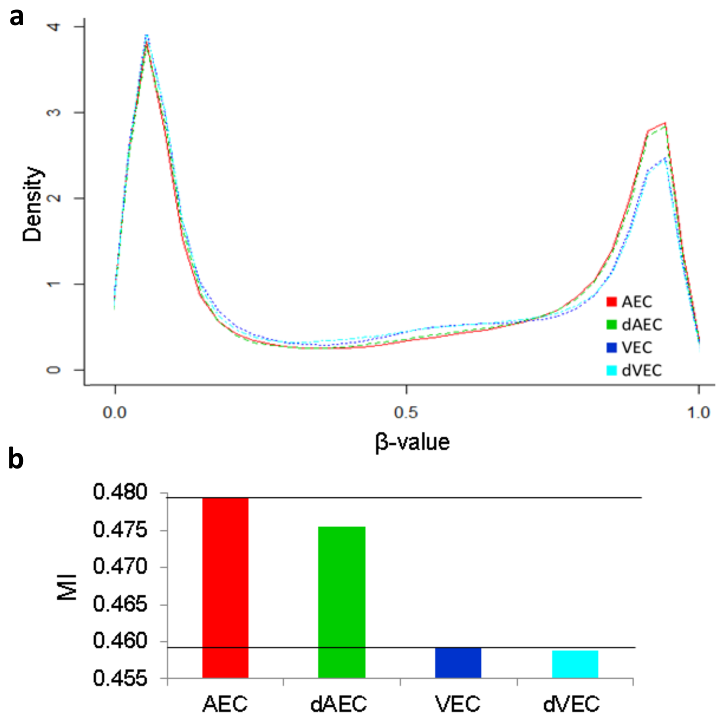

ESM Figure 4. (a) Kernel density plot representing the distribution of the averaged methylation values (β-values) of control and GDM exposed AEC and VEC, respectively. Kernel density plot showed typical bimodal distribution of β-values for both, controls and diabetic samples. (b) Methylation index (MI) was calculated for each group by calculating the mean of all Infinium β-values for that sample. The MIs were then grouped by cell type and disease.

ESM Fig. 5

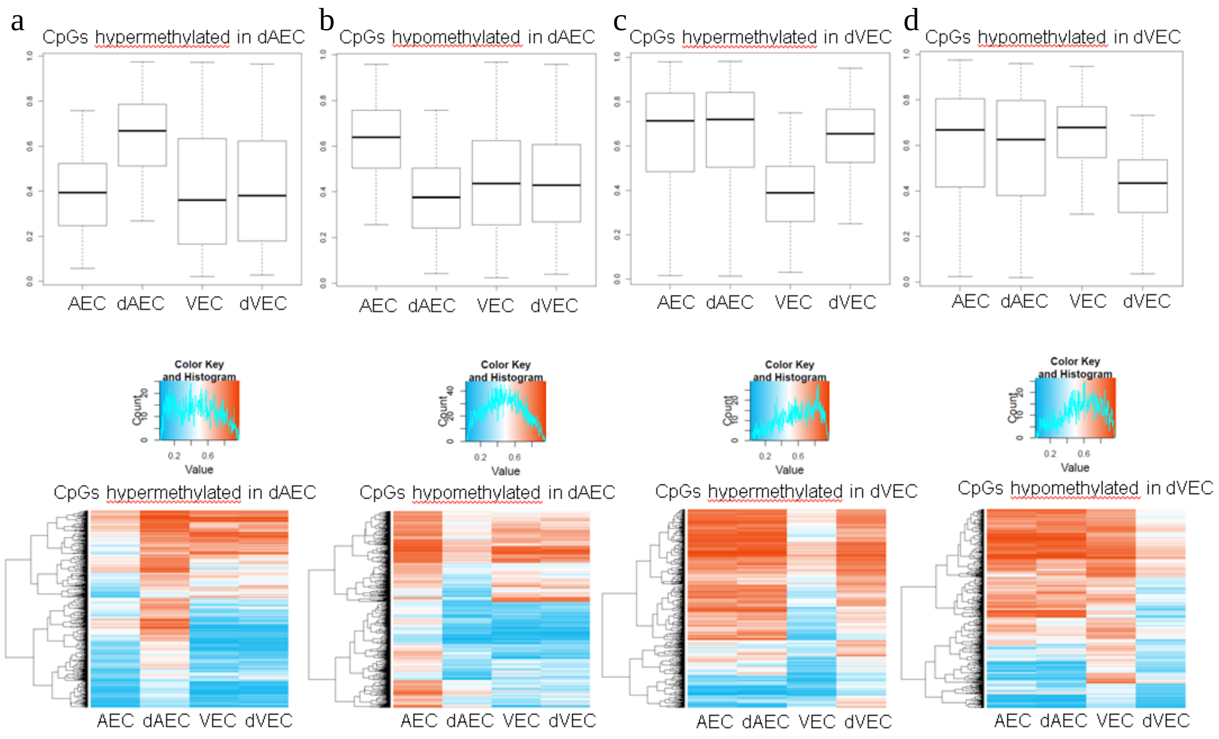

ESM Figure 5. Box plots and heat maps of methylation levels in control (AEC, VEC) and GDM exposed (dAEC, dVEC) feto-placental endothelial cells. Methylation changes are represented as CpGs hypermethylated (a) and hypomethylated (b) in dAEC, and as CpGs hypermethylated (c) and hypomethylated (d) in dVEC.

ESM Fig. 6

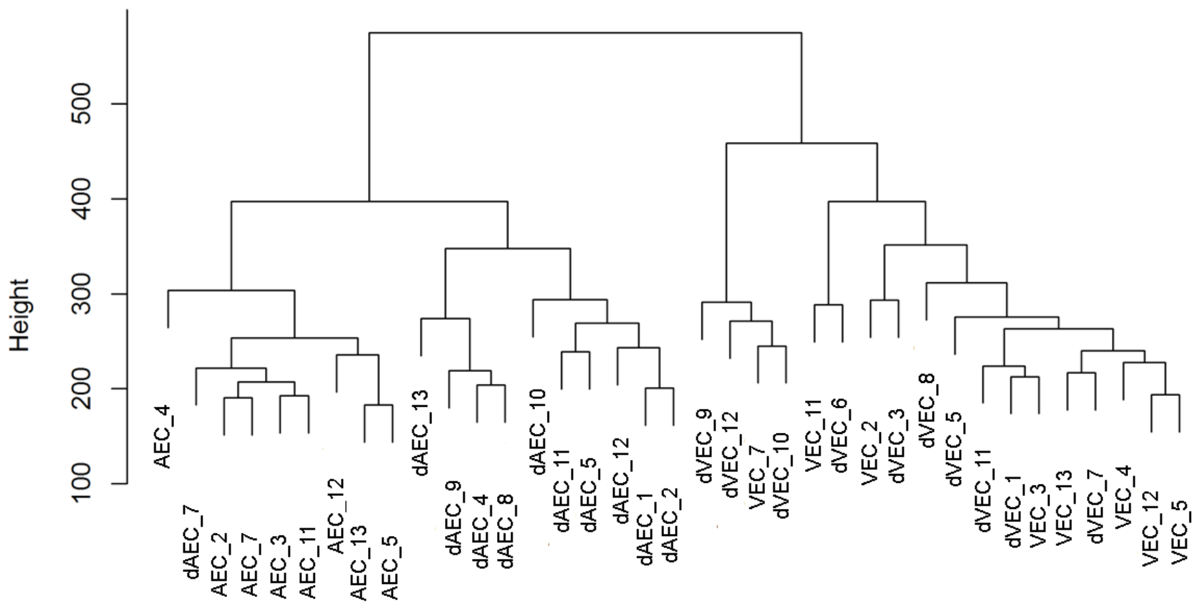

ESM Figure 6. Cluster dendrogram of expression arrays showing influence of cell type and GDM on sample clustering.
